# Discovering structural motifs using a structural alphabet: Application to magnesium-binding sites

**DOI:** 10.1186/1471-2105-8-106

**Published:** 2007-03-28

**Authors:** Minko Dudev, Carmay Lim

**Affiliations:** 1 Institute of Biomedical Sciences, Academia Sinica, Taipei 115, Taiwan; 2 Department of Chemistry, National Tsing-Hua University, Hsinchu 300, Taiwan

## Abstract

**Background:**

For many metalloproteins, sequence motifs characteristic of metal-binding sites have not been found or are so short that they would not be expected to be metal-specific. Striking examples of such metalloproteins are those containing Mg^2+^, one of the most versatile metal cofactors in cellular biochemistry. Even when Mg^2+^-proteins share insufficient sequence homology to identify Mg^2+^-specific sequence motifs, they may still share similarity in the Mg^2+^-binding site structure. However, no structural motifs characteristic of Mg^2+^-binding sites have been reported. Thus, our aims are (i) to develop a general method for discovering structural patterns/motifs characteristic of ligand-binding sites, given the 3D protein structures, and (ii) to apply it to Mg^2+^-proteins sharing <30% sequence identity. Our motif discovery method employs structural alphabet encoding to convert 3D structures to the corresponding 1D structural letter sequences, where the Mg^2+^-structural motifs are identified as recurring structural patterns.

**Results:**

The structural alphabet-based motif discovery method has revealed the structural preference of Mg^2+^-binding sites for certain local/secondary structures: compared to all residues in the Mg^2+^-proteins, both first and second-shell Mg^2+^-ligands prefer loops to helices. Even when the Mg^2+^-proteins share no significant sequence homology, some of them share a similar Mg^2+^-binding site structure: 4 Mg^2+^-structural motifs, comprising 21% of the binding sites, were found. In particular, one of the Mg^2+^-structural motifs found maps to a specific functional group, namely, hydrolases. Furthermore, 2 of the motifs were not found in non metalloproteins or in Ca^2+^-binding proteins. The structural motifs discovered thus capture some essential biochemical and/or evolutionary properties, and hence may be useful for discovering proteins where Mg^2+ ^plays an important biological role.

**Conclusion:**

The structural motif discovery method presented herein is general and can be applied to any set of proteins with known 3D structures. This new method is timely considering the increasing number of structures for proteins with unknown function that are being solved from structural genomics incentives. For such proteins, which share no significant sequence homology to proteins of known function, the presence of a structural motif that maps to a specific protein function in the structure would suggest likely active/binding sites and a particular biological function.

## Background

Magnesium is one of the most versatile metal cofactors in cellular biochemistry, serving both intra and extracellular, catalytic and/or structural roles [1]. It is used to stabilize a variety of protein structures; e.g., the interface of the ribonucleotide reductase subunits [2]. It is also used to stabilize nucleic acids by alleviating electrostatic repulsion between negatively charged phosphates. Furthermore, Mg^2+^, together with Ca^2+^, stabilize biological membranes by charge neutralization after binding to the carboxylated and phosphorylated headgroups of lipids. It also activates enzymes that regulate the biochemistry of nucleic acids such as restriction nucleases, ligases, and topoisomerases, and is essential for the fidelity of DNA replication [1]. Divalent Mg^2+ ^is a "hard" ion and prefers "hard" ligands of low polarizability like oxygen. It tends to bind directly to the amino acid residues, primarily to the Asp/Glu carboxylic side chains, followed by the Asn/Gln side chains or peptide backbone carbonyl groups [3]. The rest of the metal coordination sphere, which is usually octahedral, is complemented by water ligand(s).

Unlike Zn^2+ ^and Ca^2+^-binding sites, only a few, relatively short, sequence motifs have been discovered for Mg^2+ ^proteins with close sequence homology. These include the -NA**D**F**D**G**D**- motif, found in different RNA polymerases, DNA Pol I and HIV reverse transcriptase, and the -YX**DD- **or -LX**DD**- motifs in reverse transcriptase and telomerase, where residues in bold are the Mg^2+ ^ligands [4]. As the known Mg^2+ ^sequence motifs are short, they could easily be found in other nonMg^2+^-proteins and would *not *be expected to be Mg^2+^-specific. Interestingly, some homology in the 3D structure of the Mg^2+^-binding sites has been observed for certain classes of enzymes such as restriction enzymes, bacterial and viral RNase H domains, and viral integrases [4]. However, systematic studies of the structural preference/conservation of Mg^2+^-binding sites in nonhomologous proteins have not been reported; hence, no structural motifs of the Mg^2+^-binding sites have been extracted.

The aims in this work are to address the following intriguing questions: (1) Do Mg^2+^-binding sites exhibit any preference for certain local/secondary structures? If so, which types of local/secondary structures are favored and which are disfavored? (2) Even when the Mg^2+^-proteins share no significant sequence homology, do they share a similar Mg^2+^-binding site structure? (3) If structural motifs of the Mg^2+^-binding sites exist, do they map to specific protein functions? (4) Are the structural motifs Mg^2+^-specific? In particular, are they found in proteins that do not bind metal ions or in proteins that bind Ca^2+^, which like Mg^2+^, is also a divalent "hard" ion, binding preferentially to "hard" oxygen-containing ligands?

To address the aforementioned questions, we have developed a general strategy for discovering 3D motifs that are hidden in the local structure of the active/binding site, based on the fact that the local structure is generally more evolutionary conserved than the amino acid sequence [5]. The 3D motifs of the metal-binding sites were obtained by encoding the 3D representation based on Cartesian coordinates into a 1D representation based on a 16-letter structural alphabet [6,7]. The structural alphabet represents recurring short structural prototypes and has been used to (i) compare/analyze 3D structures [8-10], (ii) predict protein 3D structures from amino acid sequences [6,11], (iii) reconstruct the protein backbone [12], and (iv) model loops [13]. However, it has not been used to discover structural motifs of metal/ligand-binding sites in proteins. First, the structural-alphabet based motif discovery approach was validated by using it to "rediscover" the structural motif of Cys_4 _Zn-finger domains, which are known to adopt a specific structure. Next, it was used to discover structural motifs of Mg^2+^-binding sites in a set of nonredundant Mg^2+^-proteins sharing <30% sequence identity. The results reveal clear trends in the structural composition of Mg^2+^-binding sites, 4 Mg^2+^-structural motifs, and important relationships between these motifs and other features of the proteins. The specificity of the structural motifs discovered for certain Mg^2+^-binding sites was assessed by determining their occurrence in a set of nonredundant non-metal containing protein structures and in a set of nonredundant Ca^2+^-bound protein structures.

## Results

### Validation against Proteins with known Structural Motifs

**Figure 1:**
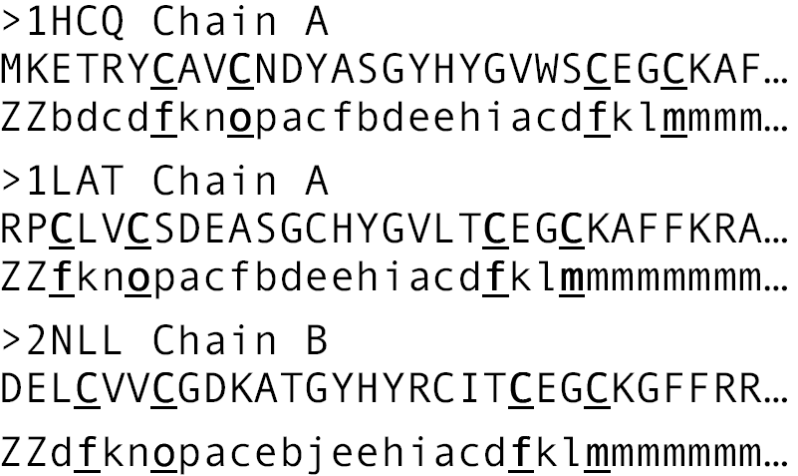
**Zn-binding site structural motifs derived from the structural alphabet representation of 3 Zn-finger proteins**. For each protein, the PDB entry and chain is given, followed below by its amino acid sequence (in capital letters), aligned with the corresponding structural alphabet representation (lower-case letters); '*Z*', means a letter cannot be assigned to this residue (see Methods). Zn^2+^-binding residues are underlined and in bold. Only the first 30 amino acid residues are shown. The C_α _root-mean-square deviation RMSD of 1LAT and 2NLL from 1HCQ are 1.73 and 1.33 Å, respectively, whereas that of 1LAT from 2NLL is 1.25 Å.

To test the structural alphabet-based strategy for discovering metal-binding site structural motifs, a database of 42 structural zinc sites from 29 proteins in previous work [14] was searched for proteins containing the **C**(2)**C**(13–15)**C**(2)**C **sequence motif, where the number in parentheses indicates the number of amino acid residues separating the conserved Zn-binding cysteines. Proteins with such a sequence motif belong to the Zn-finger family of the nuclear receptor type, having a Cys_4 _Zn-binding site [15], which is known to adopt a specific structure. Each of the Zn-proteins containing the **C**(2)**C**(13–15)**C**(2)**C **sequence motif was represented by a 1D structural alphabet, as described in Methods and illustrated in Figure 1. All of these proteins were found to possess a *f(2)o(13–15)f(2)m *structural motif of the Zn-binding site (see Figure 1). This shows that the structural-alphabet based approach for discovering new structural motifs seems promising.

### Structural Preference of Mg^2+^-Binding Sites

**Figure 2:**
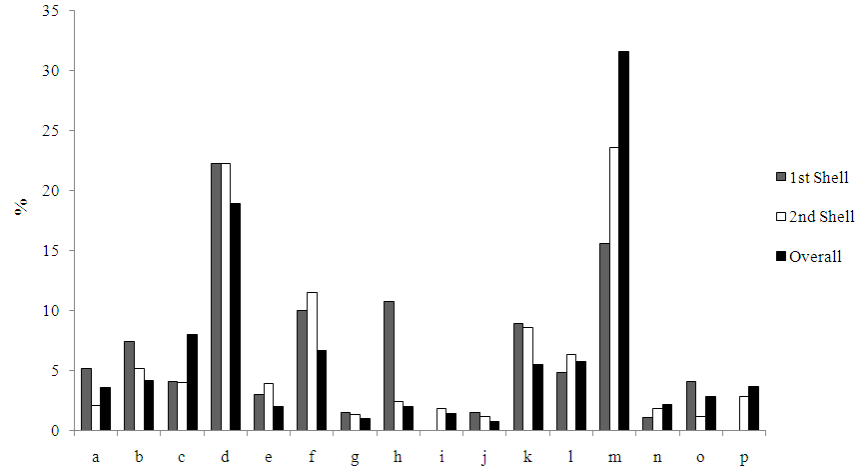
**The percentage letter frequency distributions of first-shell amino acid residues (gray), second-shell amino acid residues (white), and all amino acid residues (black) in the Mg^2+^-proteins**. There is a total of 25,406 amino acid residues in the Mg^2+^-proteins, of which 250 are in the first shell, while 898 are in the second shell

Although the 70 Mg^2+^-proteins used herein share <30% sequence identity, do their Mg^2+^-binding sites prefer certain local structures? To answer this question, the 3D structure of each of the 70 nonredundant Mg^2+ ^proteins was represented by a 16-letter structural alphabet (see Methods and Additional file 1), and the frequencies of the letters in all the first-and second-shells as well as in the entire Mg^2+ ^dataset were compared (see Figure 2). The results reveal a clear preference towards certain types of local structures in the Mg^2+^-binding sites. The '*b'*, *'d'*, *'f'*, and *'h' *frequencies of first-shell Mg^2+^-ligands and the '*d', 'e', 'f' *and *'k' *frequencies of second-shell Mg^2+^-ligands are statistically significantly higher than the respective frequencies of all the amino acid residues in the dataset (see Table 1). Both first and second-shell Mg^2+^-ligands favor the '*d*' and '*f' *structures. Furthermore, the first-shell (but not the second-shell) Mg^2+^-ligands *strongly *prefer the local structure *'h'*, whose frequency of first-shell ligands is 5.3-fold greater than that of all residues in Mg^2+ ^proteins. However, compared to all amino acid residues in the Mg^2+ ^proteins, both first and second-shell Mg^2+^-ligands disfavor certain local protein structures such as the '*c*' and '*m*' structures: The '*c', 'i', 'm'*, and *'p' *frequencies of first-shell Mg^2+^-ligands and the '*a', 'c', 'm' *and *'o' *frequencies of second-shell Mg^2+^-ligands are statistically significantly lower than the respective frequencies of all the amino acid residues in the dataset (see Table 1).

**Table 1:** The letter and secondary structural element (SSE) frequency distributions and 2-sample T-tests of first-and second-shell amino acid residues vs. all amino acid residues in the Mg^2+^-proteins

	1^st^-shell vs. all residues			2^nd^-shell vs. all residues		
Letter, *x*^a^	ν_*x*,1_/ν_*x*, all_^b^	T-test^c^	p-value^c,d^	ν_*x*,2_/ν_*x*, all_^e^	T-test^c^	p-value^c,d^

*a*	1.47	1.4037	0.0802	0.57	2.4731	**0.0067**
*b*	1.86	2.7909	**0.0027**	1.20	1.2200	0.1113
*c*	0.56	2.0160	**0.0219**	0.50	4.3510	**<0.0001**
*d*	1.23	1.7376	**0.0412**	1.23	3.1829	**0.0008**
*e*	1.46	1.0111	0.1560	2.03	4.1825	**<0.0001**
*f*	1.47	1.9389	**0.0263**	1.70	5.4060	**<0.0001**
*g*	1.15	0.2494	0.4015	1.18	0.5381	0.2953
*h*	5.29	9.3752	**< 0.0001**	1.19	0.7921	0.2142
*i*	0	1.8928	**0.0292**	1.34	1.1910	0.1168
*j*	2.21	1.6156	0.0531	1.54	1.3401	0.0901
*k*	1.40	1.4992	0.0669	1.60	4.1820	**<0.0001**
*l*	0.76	0.9209	0.1786	1.08	0.5978	0.275
*m*	0.52	2.9377	**0.0017**	0.74	5.2192	**<0.0001**
*n*	0.53	1.1306	0.1291	0.88	0.5208	0.3013
*o*	1.52	1.4066	0.0798	0.35	3.3637	**0.0004**
*p*	0	3.1174	**0.0009**	0.77	1.3204	0.0934
SSE, *x*						
Loop	1.56	2.5575	**0.0053**	1.47	2.1874	**0.0144**
β-strands	1.30	1.0780	0.1405	1.34	1.2170	0.1118
α-helices	0.47	3.6454	**0.0002**	0.51	3.3621	**0.0004**

^a^16-letter structural alphabet defined by de Brevern and co-workers (see Methods and original reference) [6]. ^b^The ratio of the letter/SSE '*x*' frequency of first-shell amino acid residues to that of all amino acid residues in the 70 Mg^2+ ^proteins. ^c^The statistical analyses were carried out using the package, SAS/STAT version 8 (SAS Institute, NC). ^d^P-values <0.05 are highlighted in bold. ^e^The ratio of the letter/SSE '*x*' frequency of second-shell amino acid residues to that of all amino acid residues in the 70 Mg^2+ ^proteins.

**Figure 3:**
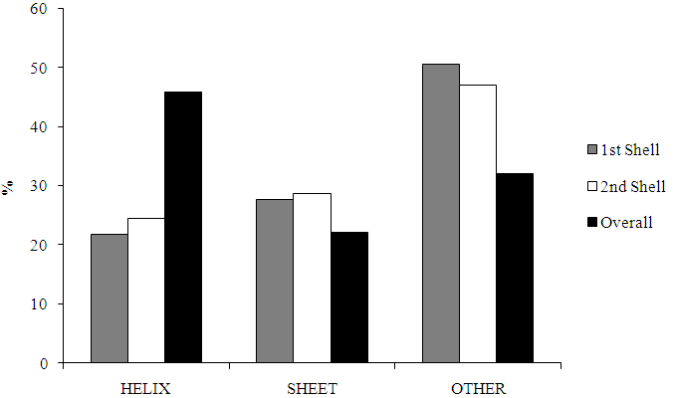
**The percentage secondary structure frequency distributions of first-shell amino acid residues (gray), second-shell amino acid residues (white), and all amino acid residues (black) in the Mg^2+^-proteins**. The amino acid residues found in α-helices, β-strands, or loops are according to the secondary structure information in the PDB files.

To relate the observed bias of the first-shell Mg^2+^-ligands for *certain *structures to standard regular and irregular secondary structures, the percentage frequency distribution of first-shell, second-shell, and all amino acid residues that are found in α-helices, β-strands, or loops in the Mg^2+^-proteins according to the secondary structure information in the Protein Data Bank [16] (PDB) files were computed (see Figure 3). The *loop *occurrence frequency of the first or second-shell Mg^2+^-residues (47–50%) is significantly higher than that of all residues (~32%) with p-values ≤ 0.014 (see Table 1). However the *β-sheet *occurrence frequency of the first or second-shell Mg^2+^-residues (~29%) is *not *significantly higher than that of all residues (~22%). In contrast, the α-*helix *occurrence frequency of the first or second shell Mg^2+^-residues (22–23%) is nearly half of the respective frequency of all residues (~46%) with p-values ≤ 0.0004.

In summary, the Mg^2+^-binding sites generally prefer certain local structures: compared to all amino acid residues in the Mg^2+ ^proteins, both first and second-shell ligands tend to prefer loops to helices. This may be due to the need to position not only the first and second-shell ligands, but also the helix dipole, in a proper orientation for metal binding.

### Structural Motifs of Mg^2+^-Binding Sites

**Figure 4:**
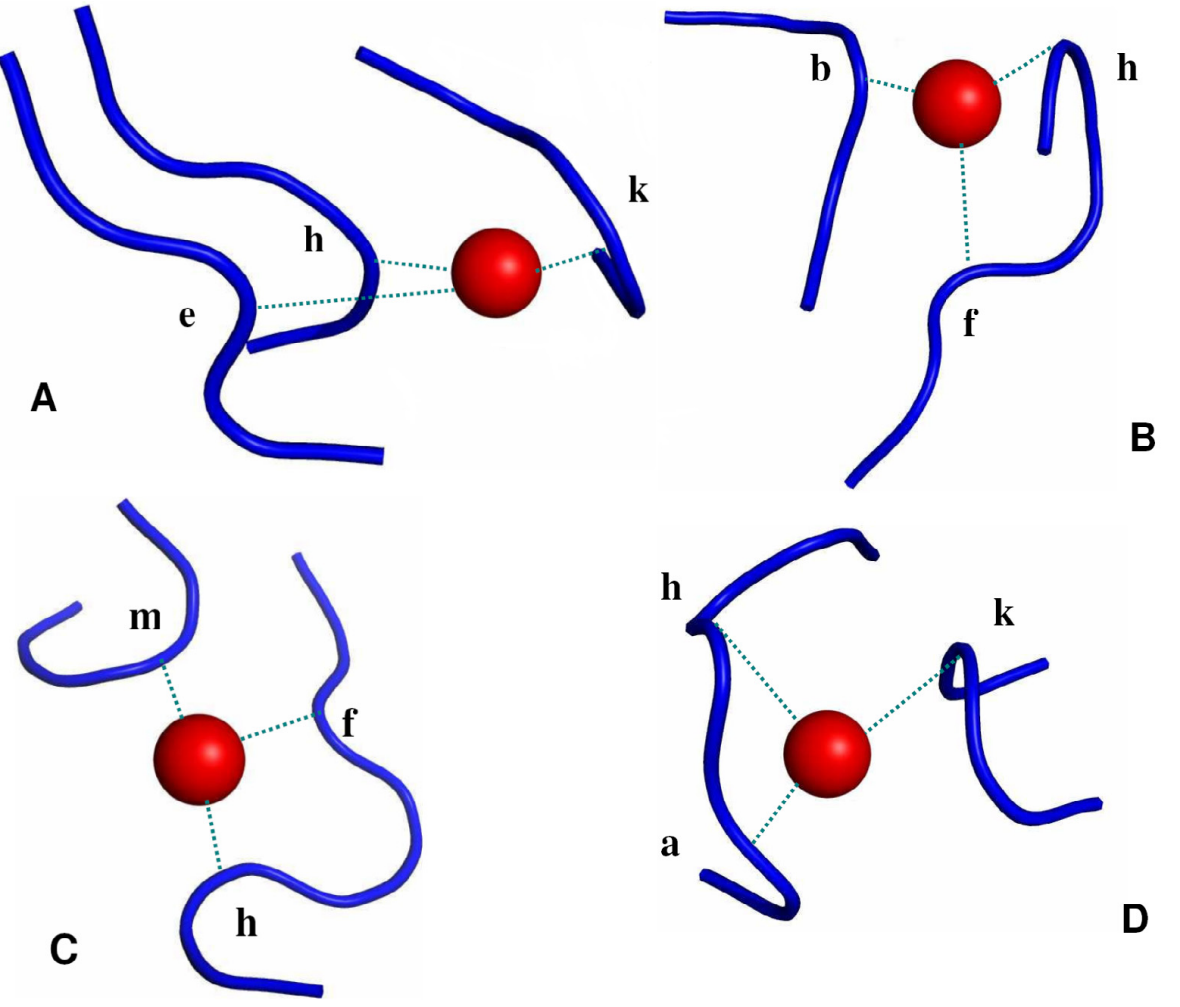
**The conserved local structures of the 4 Mg^2+^-structural motifs**. (a) *e(24–47)h(24)k*, (b) *f(1)h(109–349)b*, (c) *f(2)h(126–158)m*, and (d) *k(26–29)h(1)a*.

Even when the Mg^2+^-proteins share no significant sequence homology (<30% sequence identity), do any of them share a common structure of the metal-binding site? Such structural motifs are defined in this work to exist if 3 or more Mg^2+^-binding sites have the same first-shell letters and similar interletter spacing (see Methods and Additional file 1). These structural motifs are listed in Table 2 and illustrated in Figure 4, while first-shell structural patterns that are common to only 2 Mg^2+^-binding sites are listed in Additional file 2. For the first shell, 4 structural motifs, representing about a fifth (16/77 or 21%) of all Mg^2+^-binding sites, were discovered. All 4 motifs occur in proteins whose functions are either Mg^2+^-dependent or whose native co-factors are Mg^2+ ^according to UniProt and/or the literature. Consistent with the above finding that the *'h' *structure is preferred by the first-shell Mg^2+^-ligands, it is in the middle of all 4 motifs and the partial motif '*f(1–2)h*' accounts for half of the Mg^2+^-proteins with structural motifs. For the second shell, too many residues define the Mg^2+^-binding site; hence not enough Mg^2+^-binding sites possess the same second-shell letters and similar interletter spacing. However, 5 partial motifs for the second shell were found: These are *f(1)lm, kl(0–1)m, d(1–2)ff, d(1)e(1)i(0–5)l, f(1)l(18–25)d*, with an occurrence frequency of 21, 12, 11, 8, and 6%, respectively.

**Table 2:** 1^st^-shell structural motifs in Mg^2+^-proteins

*Motif*^*a*^	*PDB code*	*Mg^2+ ^-Ligands*	*CATH number*^*b*^	*Functional Group*^*c*^	*EC code*^*d*^
e(24–47)h(24)k	1SJC	D^189^, E^214^, D^239^	3.20.20.120	Lyase^e^, Isomerase^f^	-
	1TKK	D^191^, E^219^, D^244^	3.20.20.120	Isomerase^f^	-
	2AKZ	D^244^, E^292^, D^317^	-	Lyase^e^	4.2.1.11
					
f(1)h(109–349)b	1O08	D^1008^, D^1010^, D^1170^	3.40.50.1000	Isomerase^f^	5.4.2.6
	1U7P	D^11^, D^13^, D^123^	NYC	Hydrolase^g^	-
	1WPG	D^351^, T^353^, D^703^	3.40.50.1000	Hydrolase^g^	3.6.3.8
	2B82	D^44^, D^46^, D^167^	3.40.50.1000	Hydrolase^g^	3.1.3.2
	2C4N	D^9^, D^11^, D^201^	NYC	Hydrolase^g^	-
					
f(2)h(126–158)m	1KA1	D^142^, D^145^, D^294^	3.30.540.10	Hydrolase^g^	3.1.3.7
	1NUY	D^1118^, D^1121^, E^1280^	3.30.540.10+ 3.40.190.80	Hydrolase^g^	3.1.3.11
	2BJI	E^1090^, D^1093^, D^1220^	3.30.540.10+ 3.40.190.80	Hydrolase^g^	3.1.3.25
					
k(26–29)h(1)a	1ITZ	D^168^, N^198^, I^200^	3.40.50.970	Transferase^h^	2.2.1.1
	1POX	D^447^, N^474^, Q^476^	3.40.50.970+ 3.40.50.1220	Oxidoreductase^i^	1.2.3.3
	1UMD	D^175^, N^204^, Y^206^	3.40.50.970	Oxidoreductase^i^	1.2.4.4
	1ZPD	D^440^, N^467^, G^469^	3.40.50.970	Lyase^e^	4.1.1.1
	2C3M	D^963^, T^991^, V^993^	3.40.50.970	Oxidoreductase^i^	1.2.7.1

^a^The number in parentheses indicates the number of residues separating the letters corresponding to the Mg^2+^-bound residues. ^b^The CATH code of the domain containing the Mg^2+^-ligands; a dash implies that no domain could be assigned to the PDB entry, while NYC means the protein has not yet been chopped. ^c^The functional group from the PDB header. ^d^The enzyme class from PDBsum [25]; a dash means no EC code was found. ^e^Lyases (EC4---) catalyze C-C/O/N and other bond cleavage; e.g., RCOCOOH → RCOH + CO_2_. ^f^Isomerases (EC5---) catalyze geometric changes within a molecule. ^g^Hydrolases (EC3---) catalyze hydrolytic bond cleavage: AB + H_2_O → AOH + BH. ^h^Transferases (EC2---) catalyze AB + C → A + BC. ^i^Oxidoreductases (EC1---) catalyze oxido-reductions: AH + B → A + BH (reduced) and A + O → AO (oxidized).

Each of the 4 motifs in Table 2 is found in proteins containing Mg^2+^-binding domains belonging to the same superfamily. This is evidenced by the fact that proteins with the same Mg^2+^-structural motif have Mg^2+^-binding domains belonging to the same superfamily with the same CATH numbers (Table 2), implying structurally homologous domains. For example, all 3 proteins with the *f(2)h(126–158)m *motif possess in common a Mg^2+^-binding domain belonging to the fructose-1,6-bisphosphatase, subunit A, domain 1 superfamily (CATH number 3.30.540.10). Likewise, all 5 proteins with the *k(26–29)h(1)a *motif possess Mg^2+^-binding domains with the same CATH number, 3.40.50.970. The fact that the motifs are found in structurally homologous Mg^2+^-binding domains further supports the use of the structural alphabet to discover motifs.

**Figure 5:**
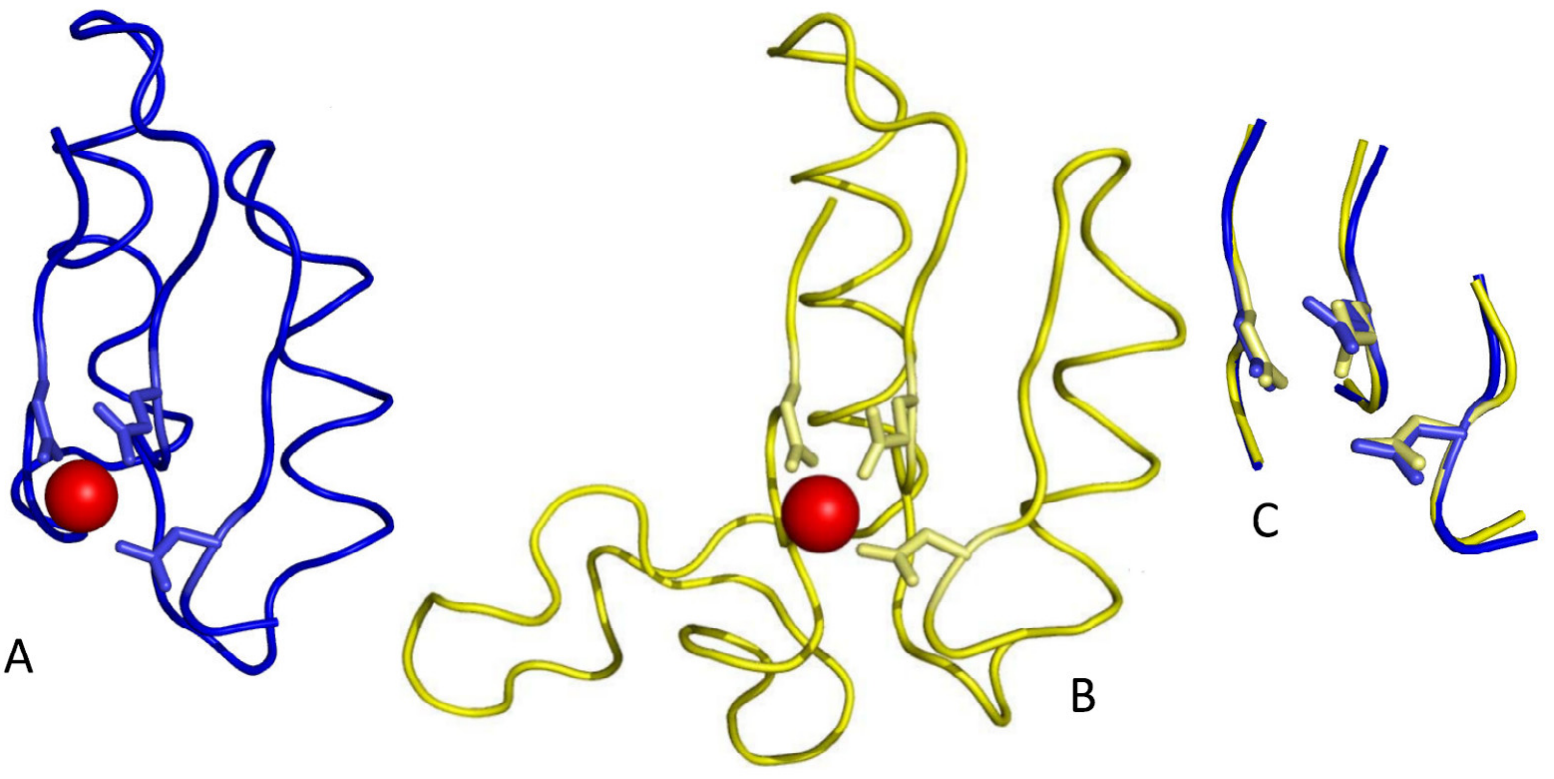
**The conserved binding site of 2 nonhomologous Mg^2+^-proteins**. (a) Cartoon diagram of the metal-binding domain in N-acylamino acid racemase (1SJC). (b) Cartoon diagram of the metal-binding domain in gamma enolase (2AKZ). (c) Superposition of the first-shell structural letters of 1SJC (blue) and 2AKZ (yellow).

The first-shell motifs discovered herein can also help to uncover relationships between proteins with unassigned CATH numbers. For example, 2 of the 3 proteins with the *e(24–47)h(24)k *motif (1SJC and 1TKK) possess Mg^2+^-binding domains pertaining to the enolase superfamily (CATH number 3.20.20.120), whereas the third protein (2AKZ) has not yet been assigned a domain and therefore has no CATH number. Although the n-acylamino acid racemase (1SJC) and gamma enolase (2AKZ) proteins do not share significant sequence homology (only 15.4% identity) and overall structure similarity (protein backbone rmsd = 17.5 Å), they possess similar Mg^2+^-binding site structures (backbone rmsd of the first-shell letters = 0.5 Å), as shown in Figure 5. In analogy, 3 of the 5 proteins with the *f(1)h(109–349)b *motif (1O08, 1WPG, and 2B82) possess Mg^2+^-binding domains with the same CATH number (3.40.50.1000), whereas the other 2 proteins (1U7P and 2C4N) have not yet been chopped into domains and therefore have not been assigned CATH numbers. The results in Table 2 predict that the Mg^2+^-dependent phosphatase (1U7P) and NagD (2C4N) proteins are likely to possess Mg^2+^-binding domains that are structurally homologous to those assigned with the CATH number 3.40.50.1000.

### Relation between Mg^2+^-Structural Motifs and PROSITE Sequence Motifs

To see if any of the Mg^2+^-proteins containing structural motifs match sequence motifs stored in the PROSITE database [17], the sequences of the proteins listed in Table 2 were scanned for the occurrence of PROSITE sequence motifs. None of the proteins match any PROSITE sequence motifs encompassing residues involved in Mg^2+^-binding. However, the halotolerance protein hal 2 (1KA1) containing the *f(2)h(126–158)m *motif matched 2 inositol monophosphatase family signatures (PROSITE PDOC00547) containing conserved metal-binding residues. This supports the '*f(2)h(126–158)m*' motif as a signature of Mg^2+^-binding sites.

### Relation between Mg^2+^-Structural Motifs and Protein Function

Do any of the structural motifs found for the Mg^2+^-proteins map to specific protein functions? To answer this question, for each of the Mg^2+^-proteins found with a structural motif, the functional group of the protein from the PDB header and enzyme classification (EC) code, if applicable, are listed in Table 2. Note that proteins belonging to the same functional group have the same first EC number. The results in Table 2 show that most of the structural motifs found for the Mg^2+^-proteins map to certain protein functions. For example, proteins with the partial *f(1–2)h *motif are all hydrolases, catalyzing the hydrolytic cleavage of mostly ester bonds (EC3.1.-.-), except for beta-phosphoglucomutase (1O08), which is an isomerase converting beta-D-glucose 1-phosphate to beta-D-glucose 6-phosphate. Interestingly, although class b acid phosphatase (2B82) and the halotolerance protein hal 2 (1KA1) contain structurally *nonhomologous *Mg^2+^-binding domains with different CATH numbers, both are phosphoric monoester hydrolases (EC3.1.3.-). Proteins with the *e(24–47)h(24)k *motif are either lyases and/or isomerases, whereas proteins with the *k(26–29)h(1)a *motif have even more diverse functions: 3 are oxidoreductases (1POX, 1UMD, 2C3M), one is a lyase (1ZPD) and the other is a transferase (1ITZ). This shows that even if the proteins share structurally homologous domains (CATH number 3.40.50.970) and structurally similar Mg^2+^-binding sites, as represented by the *k(26–29)h(1)a *motif, they can perform different functions.

### Statistical Significance of the Mg^2+^-Structural Motifs

Do the structural motifs found for Mg^2+^-proteins in Table 2 occur in other proteins that do not bind metal ions? To address this question, de Brevern's databank of protein structures that have been encoded into 1D structural sequences was searched for the occurrence of each of the 4 structural motifs listed in Table 2. Consistent with the Mg^2+ ^and Ca^2+ ^datasets, proteins in de Brevern's databank sharing ≥ 30% sequence identity with ≥ 2.5-Å resolution X-ray structures were removed. Proteins in de Brevern's databank whose structures are complexed with metal ions were also removed, yielding a set of 385 non-homologous test proteins. In order to eliminate those matched structural letters that cannot spatially bind Mg^2+^, the maximum C_α_-C_α _distance between any pair of Mg^2+^-ligands belonging to proteins of a given structural motif in Table 2 was extracted; this distance is 9.32 Å for the *e(24–47)h(24)k *motif, 8.32 Å for *f(1)h(109–349)b*, 8.44 Å for *f(2)h(126–158)m*, and 7.86 Å for *k(26–29)h(1)a*. For a given structural motif in Table 2, matched letters in the test proteins whose C_α_-C_α _distances exceed the respective maximum distance were eliminated. This resulted in no matches for the *e(24–47)h(24)k *and *f(2)h(126–158)m *motifs, whereas 2 proteins (1C3K, 1GPE) contained the *f(1)h(109–349)b *motif, and another 2 proteins (1A7U, 1JFR) contained the *k(26–29)h(1)a *motif. A check of the literature confirmed that these 4 proteins (1C3K, 1GPE 1A7U, 1JFR) do not bind metal ions. This suggests that (i) the 4 Mg^2+^-structural motifs discovered are statistically significant, and (ii) the *e(24–47)h(24)k *and *f(2)h(126–158)m *motifs could be used to predict metal-binding sites.

### Metal Preference of the Mg^2+^-Structural Motifs

To check the specificity of the 4 structural motifs in Table 2 for Mg^2+^, the same procedure used to represent the Mg^2+^-binding sites in terms of their local structure was repeated for Ca^2+^, which is the metal ion most similar to Mg^2+^. Both Mg^2+ ^and Ca^2+ ^are closed-shell divalent cations belonging to the same group (IIA) with similar chemical properties: They are both "hard" dications that prefer to bind directly to "hard" oxygen-containing anions, and are hence often found to bind in the same protein cavity [18]. Thus, the 3D structure of each of the 177 nonredundant Ca^2+ ^proteins was represented by a 16-letter structural alphabet (see Methods), and the 1D structural letter representation of the 230 Ca^2+^-binding sites are listed in Additional file 3.

None of the structural motifs in Table 2 or Additional file 2 were found in 3 or more Ca^2+^-binding sites, and therefore cannot be classified as Ca^2+^-structural motifs according to our definition. The *f(1)h(109–349)b *motif is found in the Ca^2+^-binding site of the hydrolase from the haloacid dehalogenase family (2FI1), which appears to utilize Mg^2+ ^as a natural co-factor [19]. Although the *k(26–29)h(1)a *motif is found in the calcium-binding sites of the transketolase protein (1TRK) and benzoylformate decarboxylase (1Q6Z), the latter is a Mg^2+^-dependent enzyme [20]. The *e(24–47)h(24)k *and *f(2)h(126–158)m *motifs did not match any first-shell structural letters of the Ca^2+^-binding sites, indicating that they seem to favor Mg^2+ ^over its competitor, Ca^2+^.

## Discussion and conclusion

### Comparison with Previous Structural Motif Discovery Methods

Assuming that similarity in the local active site structure implies similarity in biological function, 3D patterns/templates of key active sites have been used to suggest the function of a protein whose structure is known. The 3D patterns/templates have been constructed either manually or automatically using various methods, which have been reviewed recently by Watson et al. [21]. Recently, 3D templates in the absence of experimental data have been constructed using the evolutionary trace method to identify evolutionarily important, solvent accessible residues that cluster in the protein structure [22]. Furthermore, structural motifs for the metal-binding sites of 3 distinct metalloenzymes families; viz., DNase 1 homologs, dimetallic phosphatases, and dioxygenases, have been obtained by first identifying physical chemical property-based sequence motifs in homologous protein sequences, and subsequently identifying those motifs whose structures are conserved in members of a family/superfamily [23,24]. However, to the best of our knowledge, 3D patterns of key active sites and recurrent patterns (structural motifs) have not been identified using the structural alphabet to convert 3D structures to the respective 1D letter sequences. Also, systematic studies of the structural preference or conservation of Mg^2+^-binding sites in nonhomologous proteins and Mg^2+^-specific structural motifs have not been reported.

### Advantages of the Structural-Alphabet Based Motif Discovery Approach

This work presents the first application of the structural alphabet approach to define the 3D patterns of metal active sites and to identify recurrent patterns (structural motifs). The method requires as input only the 3D protein structure to define a 1D structural representation of the respective active site. The structural alphabet-based approach has several advantages: (i) It is efficient at handling many structures as it takes less than a minute on a present-day PC to convert a 3D structure to the corresponding 1D structural sequence. (ii) It requires no sequence comparisons, no parameters or scoring functions and can thus produce consistent structural motifs, whose structures are readily visualized, as illustrated in Figures 4 and 5. (iii) It is general and can be used to define 3D patterns not only in metal-binding sites, but also in enzyme active sites, ligand-binding clefts and interacting regions between proteins and their respective partners. The 3D patterns/motifs discovered could be incorporated in methods to detect metal/ligand-binding sites to improve their prediction accuracy.

### Secondary Structure Preference of Mg^2+^-Binding Residues

In this work, the structural alphabet-based approach has been used to reveal the structural preference of Mg^2+^-binding sites. Even though helix-like segments represented by the letter 'm' is the most common building block of the Mg^2+^-proteins in the dataset, residues that bind Mg^2+ ^disfavor helices, but favor loops. The similarity in the structural preference of the first and second-shell residues supports previous conclusions regarding the relationship between these 2 layers; namely, the structure and properties of the 2^nd- ^shell are dictated by those of the 1^st ^layer [14].

### Similar Mg^2+^-Binding Site Structures in Dissimilar Protein Sequences

The motif discovery method herein has revealed 4 structural motifs, comprising 21% of the Mg^2+^-binding sites. The 3D structural motifs discovered seems to have more predictive utility in identifying Mg^2+^-binding sites than 1D sequence motifs: A scan of the Mg^2+^-protein sequences in our dataset for the occurrence of sequence motifs stored in the PROSITE [17] database yielded only a single positive match, 1WC1, which contains a PROSITE sequence motif predicting the protein to bind Mg^2+^. However, the ScanProsite results did not identify any of the Mg^2+ ^proteins with structural motifs.

### Functional Preference of the Mg^2+^-Structural Motifs

The structural motifs discovered generally relate to the biological role of Mg^2+ ^and the function of the respective proteins. They capture some essential biochemical and/or evolutionary properties, as proteins found to contain a specific structural motif possess structurally homologous Mg^2+^-binding domains, even though they share no significant sequence homology. Furthermore, the *f(2)h(126–158)m *motif maps to a specific functional group, namely, hydrolases, indicating the apparent importance of the local Mg^2+^-binding site structure for the function of these hydrolases. As the *f(2)h(126–158)m *motif was *not *found in non-metalloproteins and in Ca^2+^-binding proteins, the presence of this motif in a novel protein structure may suggest a likely Mg^2+^-binding site and the protein function. On the other hand, the other 3 motifs map to more than one functional group, suggesting that the local Mg^2+^-binding site structure is *not *the only determinant of the protein's function.

### Why Mg^2+^-Specific Structural Motifs are Not Found For Most Mg^2+^-Proteins

Out of the 70 nonhomologous Mg^2+^-proteins, only 16 have first-shell structural motifs, while the rest do not seem to possess any metal-binding site structural motifs-why? One reason might be that some Mg^2+^-structural motifs may have been missed out in this work. As the dataset employed only proteins with Mg^2+^-bound structures (see Database subsection below), some PDB structures complexed with heavier metal ions such as Mn^2+ ^may in fact correspond to native Mg^2+^-binding site(s); moreover, not all structures of proteins whose native co-factor is Mg^2+ ^have been solved. A second reason is that for native Mg^2+^-binding sites that can accommodate other metal ions such as Ca^2+ ^or Mn^2+^, the binding-site structure need not be conserved in order to recognize a specific metal co-factor. A third reason is that although Mg^2+ ^occupied the binding site in the 3D structure, it is not the native cofactor. Although all 70 proteins are bound to Mg^2+ ^in our dataset, according to PDBSUM [25] and from the UniProt annotation and references therein, 14 proteins do not employ Mg^2+ ^as the native co-factor, while for 6 proteins, the native metal-cofactor is apparently not known (see Additional file 1). For example, calbindin d9K is a vitamin D-dependent calcium-binding protein, but in the 1IG5 structure, the native cofactor Ca^2+ ^has been replaced by Mg^2+^. In Mg^2+^-proteins with multiple Mg^2+^-binding sites, one or more sites may be non-native, as they have been artificially induced by the high Mg^2+ ^concentration used during crystallization. In these cases, the local structure of the non-native metal-binding site would not be expected to share any similarity with that of a native Mg^2+^-binding site, where the conserved local structure (as in the *f(2)h(126–158)m *motif) plays an important role in the protein's function. The absence of structural motifs for non-native Mg^2+^-binding sites indirectly supports our strategy.

## Methods

### Database

A set of 70 nonredundant Mg^2+ ^proteins was created by searching the PDB [16] for structures with resolution < 2.5-Å containing Mg^2+ ^with <30% sequence identity. Structures with residues missing in the middle of the sequence were excluded because such gaps in the structure could alter the spacing in the binding-site motifs (see below). Structures with Mg^2+ ^bound to <3 protein ligands were also excluded, as 2-residue motifs cannot be considered specific enough for any practical use. The resulting Mg^2+ ^dataset comprise 77 binding sites in 70 proteins. Note that although most Mg^2+^-proteins have only one binding site, some proteins have more than one Mg^2+^-binding sites (PDB entries 1MXG, 1NUY, 1VCL, 1WL6, 2BJI, and 2BVC). A set of nonredundant Ca^2+ ^proteins was created following the same procedure used to create the Mg^2+ ^dataset. This resulted in 230 Ca^2+^-binding sites in 177 proteins. The PDB entries, EC code, and amino acid residues bound to the metal ion in the 77 Mg^2+ ^and 230 Ca^2+ ^sites are given in Additional files 1 and 3, respectively.

### The Structural Alphabet

Each metalloprotein structure was encoded into its 1D structural sequence according to the original structural alphabet defined by de Brevern and co-workers [6]. We refer the reader to the original work [6] for details of how this alphabet was devised, and briefly outline the procedure here. The backbone of each protein from a nonredundant protein structure database was represented by consecutive 5-residue segments, each described by a vector of 8 backbone dihedral angles ***V***(ψ_n-2_, φ_n-1_, ψ_n-1_, φ_n_, ψ_n_, φ_n+1_, ψ_n+1_, φ_n+2_). The dissimilarity between 2 vectors ***V***_1 _and ***V***_2 _of dihedral angles is measured by the root-mean-square deviations of the dihedral angle values (rmsda), which is defined as the Euclidean distance among the 4 links:

rmsda (V1,V2)=∑i=14[ψi(V→1)−ψi(V→2)]2+[φi+1(V→1)−φi+1(V→2)]28
 MathType@MTEF@5@5@+=feaafiart1ev1aaatCvAUfKttLearuWrP9MDH5MBPbIqV92AaeXatLxBI9gBaebbnrfifHhDYfgasaacH8akY=wiFfYdH8Gipec8Eeeu0xXdbba9frFj0=OqFfea0dXdd9vqai=hGuQ8kuc9pgc9s8qqaq=dirpe0xb9q8qiLsFr0=vr0=vr0dc8meaabaqaciaacaGaaeqabaqabeGadaaakeaacqqGYbGCcqqGTbqBcqqGZbWCcqqGKbazcqqGHbqycqqGGaaicqGGOaakieWacqWFwbGvdaWgaaWcbaGaeGymaedabeaakiabcYcaSiab=zfawnaaBaaaleaacqaIYaGmaeqaaOGaeiykaKIaeyypa0ZaaOaaaeaadaWcaaqaamaaqahabaGaei4waSLaeqiYdK3aaSbaaSqaaiabdMgaPbqabaGccqGGOaakdaWhcaqaaiabdAfawbGaay51GaWaaSbaaSqaaiabigdaXaqabaGccqGGPaqkcqGHsislcqaHipqEdaWgaaWcbaGaemyAaKgabeaakiabcIcaOmaaFiaabaGaemOvayfacaGLxdcadaWgaaWcbaGaeGOmaidabeaakiabcMcaPiabc2faDnaaCaaaleqabaGaeGOmaidaaaqaaiabdMgaPjabg2da9iabigdaXaqaaiabisda0aqdcqGHris5aOGaey4kaSIaei4waSLaeqOXdy2aaSbaaSqaaiabdMgaPjabgUcaRiabigdaXaqabaGccqGGOaakdaWhcaqaaiabdAfawbGaay51GaWaaSbaaSqaaiabigdaXaqabaGccqGGPaqkcqGHsislcqaHgpGzdaWgaaWcbaGaemyAaKMaey4kaSIaeGymaedabeaakiabcIcaOmaaFiaabaGaemOvayfacaGLxdcadaWgaaWcbaGaeGOmaidabeaakiabcMcaPiabc2faDnaaCaaaleqabaGaeGOmaidaaaGcbaGaeGioaGdaaaWcbeaaaaa@764B@

Using an unsupervised cluster analyzer based on the above rmsda of the segments, 16 letters (also called protein blocks) were identified, which in turn comprise the structural 'alphabet'.

### Converting 3D Structure to 1D Structural Alphabet

**Figure 6:**
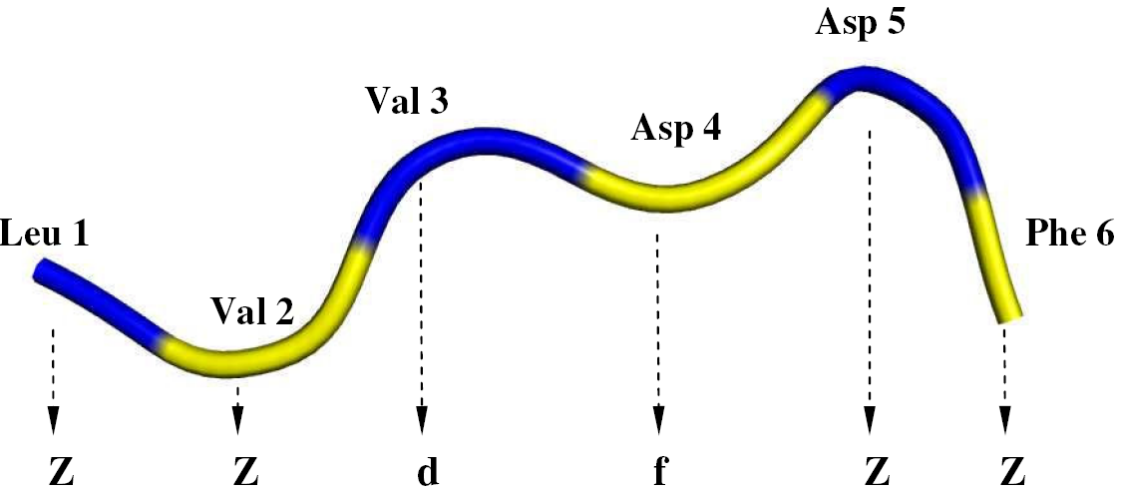
**Conversion of the 3D protein backbone into a 1D structural alphabet representation**. The first 2 and the last 2 residues are assigned '*Z*', meaning a letter cannot be assigned at these residues. The first valid assignment is '*d*', at Val 3 and spanning residues 1 to 5. The next is assigned to Asp 4 and spans residues 2 to 6.

The 3D structures of the 70 Mg^2+ ^and 177 Ca^2+ ^proteins were converted into strings of structural letters using the program PBE published in ref. 9. For a given *n- *residue protein, *n-4 *letter assignments were obtained by scanning the protein sequence using a 5-residue sliding window. The structure of each 5-residue segment is compared with that of each of the 16 letters and the letter that has the closest structure (as measured by the rmsda) to the 5-residue segment is assigned to the middle residue of that segment. This process is illustrated in Figure 6: The first letter is assigned to the 3^rd ^residue, Val, representing the first 5-residue segment. Its structure is closest to that of the structural letter '*d'*, therefore Val 3 is assigned *'d'*. Note that no letters can be assigned to the first 2 and last 2 residues of each protein.

### Definition of 1^st ^and 2^nd^-Shell Metal Ligands

Analyses of high-resolution X-ray structures with crystallographic *R *factor ≤ 0.065 of small metal complexes in the Cambridge Structural Database [26] have shown that the mean 1^*st*- ^shell Mg-O_water_, Mg-O_carboxylate_, and Mg-O_alcohol _distances do not exceed 2.11 Å, while the Ca-O_water_, Ca-O_carboxylate_, Ca-O_alcohol_, and Ca-N_imidazole _bond distances do not exceed 2.55 Å [27]. To account for the lower resolution of the PDB structures, a slightly larger cutoff was used to locate the 1^st^-shell amino acid ligands. Thus, the Mg^2+ ^and Ca^2+ ^ligands were defined as residues with a donor atom within 2.5 Å and 2.7 Å from the metal ion, respectively. The heavy atoms of the metal-bound amino acid residues were then selected as centers to search for the 2^nd^-shell protein ligands using a hydrogen-bonding cutoff of 3.5 Å [28]. Note that water molecules in the first and second shells were not identified, as they were not used to define a structural motif.

### Definition of 1^st ^and 2^nd^-Shell Structural Representation/Pattern

Since the 3D structure of each metalloprotein has been converted into the respective 1D letter sequence as described above, the letters that correspond to the metal-bound amino acid residues yielded a structural representation of the first-shell, as shown in the last columns of Additional files 1 and 3 for each metal-binding site. For example, in the case of the human/chicken estrogen receptor (1HCQ), the letters corresponding to the Zn-binding Cys residues at position 7, 10, 24 and 27 are *f, o, f*, and *m*, respectively, yielding a *f(2)o(13)f(2)m *representation of the first-shell for 1HCQ (see Figure 1).

### Definition of Structural Motifs

In previous work [29], all values of *k *between 2 and 20 were used to define a structural motif, where *k *is the number of first-shell structural patterns with the same structural letters and similar interletter spacing. Here, *k *≥ 3 was used to define a structural motif. Thus, if 3 or more proteins possess first-shell structural patterns with the same structural letters and similar interletter spacing, these proteins are assumed to share a common structural motif. For example, transketolase (1ITZ), pyruvate oxidase (1POX), 2 oxo-acid dehydrogenase alpha subunit (1UMD), pyruvate decarboxylase (1ZPD), and pyruvate-ferredoxin oxidoreductase (2C3M) share the first-shell structural pattern, *k(26–29)h(1)a*, which thus defines a structural motif.

## Authors' contributions

MD carried out all the calculations, including writing programs, and drafted the manuscript. CL conceived of the study, participated in its design and analysis/interpretation of data, and writing/revising the manuscript. Both authors have read and approved the final manuscript.

## Supplementary Material

Additional file 1 The Mg^2+^-dataset containing 77 metal-binding sites in 70 nonredundant Mg^2+^-proteins. A table listing the PDB entries, protein description, native metal-cofactors (if known), EC code, metal-bound amino acid residues, and first-shell structural representation of the 70 nonredundant Mg^2+^-proteins. Click here for file

Additional file 2 1^st^-shell patterns common to two Mg^2+^-proteins. A table listing 1^st^-shell structural patterns that is common to only 2 Mg^2+^-binding sites. Click here for file

Additional file 3 The Ca^2+^-dataset containing 230 metal-binding sites in 177 nonredundant Ca^2+^-proteins. A table listing the PDB entries, protein description, native metal-cofactors (if known), EC code, metal-bound amino acid residues, and first-shell structural representation of the 177 nonredundant Ca^2+^-proteins. Click here for file
