# Supplementary material for: Discovering structural motifs using a structural alphabet: Application to magnesium-binding sites
Source: BMC Bioinformatics. 2007 Mar 28;8:106. doi: 10.1186/1471-2105-8-106 (PMC1851716; doi:10.1186/1471-2105-8-106)
Supplement: Additional file 1 — The Mg2+-dataset containing 77 metal-binding sites in 70 nonredundant Mg2+-proteins. A table listing the PDB entries, protein description, native metal-cofactors (if known), EC code, metal-bound amino acid residues, and first-shell structural representation of the 70 nonredundant Mg2+-proteins. [file 1471-2105-8-106-S1.doc]

**Additional file 1**. The Mg^2+^−dataset containing 77 metal-binding sites in 70 nonredundant Mg^2+^−proteins^a^

| **PDB ID** | **Name** | **Native Co-factor** | **EC Code** | **Bound Residues** | **1^st^ Shell Structural Representation** |
| --- | --- | --- | --- | --- | --- |
| 1CHN | chemotaxis protein cheY | Mg^2+^ |  | D13, D57, N59 | b-f-d |
| 1DAK | dethiobiotin synthetase | Mg^2+^ | 6.3.3.3 | T16, D54, E115 | m-l-d |
| 1ED9 | alkaline phosphatase | Mg^2+^ | 3.1.3.1 | D51, T155, E322 | h-l-d |
| 1H1D | catechol-o-methyltransferase | Mg^2+^ | 2.1.1.6 | D141, D169, N170 | b-h-k |
| 1HUJ | inorganic pyrophosphatase | Mg^2+^ | 3.6.1.1 | D115, D120, D152 | h-d-d |
| 1HYO | fumarylacetoacetate | Mg^2+^ | 3.7.1.2 | D733, W734, K753, G756 | d-d-m-c |
| 1IDO | integrin | ? |  | S142, S144, T209 | f-l-d |
| 1IG5 | calbindin d9k | ? |  | D54, N56, D58, E60 | m-o-o-a |
| 1IQ8 | archaeosine trna-guanine transglycosylase | Zn^2+^ | 2.4.2.29 | A528, M566, I567, F569 | k-m-m-m |
| **1ITZ** | **transketolase** | **Mg^2+^** | **2.2.1.1** | **D168, N198, I200** | **k-h-a** |
| 1IV2 | 2-c-methyl-d-erythritol 2,4-cyclodiphosphate synthase | ? | 4.6.1.12 | D8, H10, H42 | d-d-m |
| 1J9J | stationary phase survival protein | Mg^2+^ | 3.1.3.5 | D8, D9, N95 | e-o-e |
| 1JYL | ctp:phosphocholine cytidylytransferase | ? |  | D107, E216, D218 | b-d-b |
| **1KA1** | **halotolerance protein hal2** | **Mg^2+^** | **3.1.3.7** | **D142, D145, D294** | **f-h-m** |
| 1KA2 | m32 carboxypeptidase | ? |  | H269, H273, E299 | m-m-m |
| 1KHZ | adp-ribose pyrophosphatase | Mg^2+^ | 3.6.1.13 | E112, E116, E164 | m-n-a |
| 1KTG | iadenosine tetraphosphate hydrolase | divalent ions? | 3.6.1.17 | E52, E56, E103 | m-n-b |
| 1MDL | mandelate racemase | Mg^2+^ | 5.1.2.2 | D195, E221, E247 | e-h-l |
| 1MXG |  | ? | 5.3.1.5 | D252, D256, I292 | m-k-k |
|  |  |  |  | D347, D349, E350 | f-d-l |
|  |  |  |  | G24, E80, E88 | l-d-m |
| 1N67 | clumping factor |  |  | N267, A269, V323 | k-c-m |
| 1NMP | hypothetical protein ybgi | divalent ions? |  | H63, D101, E219 | b-m-m |
| **1NUY** | **fructose-1,6-bisphosphatase** |  | **3.1.3.11** | E1097, D1118, L1120 | k-f-g |
|  |  | **Mg^2+^** |  | **D1118, D1121, E1280** | **f-h-m** |
| **1O08** | **beta-phosphoglucomutase** | **Mg^2+^** | **5.4.2.6** | **D1008, D1010, D1170** | **f-h-b** |
| 1OBW | inorganic pyrophosphatase | Mg^2+^ | 3.6.1.1 | D65, D70, D102 | o-d-d |
| 1OFH | atp-dependent protease hslv | ? | 3.4.25.- | G157, C160, T163 | m-c-c |
| **1POX** | **pyruvate oxidase** | **Mg^2+^** | **1.2.3.3** | **D447, D474, Q476** | **k-h-a** |
| 1PT6 | integrin alpha-1 | ? |  | S152, S154, D253 | f-l-b |
| 1Q6Z | benzoylformate decarboxylase | Mg^2+^ | 4.1.1.7 | N117, L118, R120 | m-m-c |
| 1SHQ | alkaline phosphatase | ? | 3.1.3.1 | D37, T151, E310 | h-l-d |
| **1SJC** | **n-acylamino acid racemase** | **Mg^2+^** |  | **D189, E214, D239** | **e-h-k** |
| 1T0F | transposon tn7 transposition protein | divalent ion? |  | D114, Q130, V131 | b-d-d |
| 1T1S | 1-deoxy-d-xylulose 5-phosphate reductoisomerase | divalent ion? | 1.1.1.267 | D149, E151, E230 | l-l-m |
| **1TKK** | **similar to chloromuconate cycloisomerase** | **Mg^2+^** |  | **D191, E219, D244** | **e-h-k** |
| 1TQY | beta-ketoacyl synthase/acyl transferase | ? | 2.3.1.41 | N307, A308, E355 | d-f-m |
| 1TW1 | beta-1,4-galactosyltransferase 1 | Mn^2+^ | 2.4.1.22 | D254, H344, H347 | b-d-d |
| **1U7P** | **magnesium-dependent phosphatase-1** | **Mg^2+^** |  | **D11, D13, D123** | **f-h-b** |
| **1UMD** | **2-oxo acid dehydrogenase alpha subunit** | **Mg^2+^** | **1.2.4.4** | **D175, N204, Y206** | **k-h-a** |
| 1V71 | hypothetical protein c320.14 in chromosome iii | ? | 5.1.1.- | E208, G212, D214 | d-m-k |
| 1VCL | hemolytic lectin cel-iii | ? |  | N32, I33, N72, V73, I131 | d-d-d-d-d |
|  |  | ? |  | N177, V178, N218, V219, D265, V266 | d-d-d-d-d-d |
| 1W79 | d-alanyl-d-alanine carboxypeptidase | ? | 3.4.16.4 | E188, H247, E251 | o-m-m |
| 1WC1 | adenylate cyclase | ? | 4.6.1.1 | D1017, I1018, D1061 | d-e-b |
| 1WDC | scallop myosin | Ca^2+^ |  | D28, D30, D32, F34, D39 | m-o-o-a-m |
| 1WL6 | xaa-pro aminopeptidase | Mn^2+^ |   3.4.11.9 | D271, H354, E383, E406 | d-c-d-d |
|  |  | Mn^2+^ |  | D260, D271, E406 | d-d-d |
| **1WPG** | **sarcoplasmic/endoplasmic reticulum calcium atpase 1** | **Mg^2+^** | **3.6.3.8** | **D351, T353, D703** | **f-h-b** |
| 1WQA | phospho-sugar mutase | ? | 5.4.2.8 | S101, D243, D245, D247 | j-f-o-a |
| 1WVM | alkaline serine protease | ? | 3.4.21.- | A169, Y171, V174, Q195 | l-m-c-b |
| 1WZC | mannosyl-3-phosphoglycerate phosphatase | Mg^2+^ | 3.1.3.70 | D8, D10, S169, D204 | f-h-k-b |
| 1XXX | dihydrodipicolinate synthase | ? | 4.2.1.52 | A162, H164, I167 | m-m-n |
| 1Y8A | hypothetical protein af1437 | ? |  | D5, E7, D207 | f-h-a |
| 1YIO | response regulatory protein | ? |  | D12, D55, R57 | b-d-f |
| 1YL7 | dihydrodipicolinate reductase | ? | 1.3.1.26 | V20, A23, L26 | m-c-c |
| 1YQ2 | beta-galactosidase | ? | 3.2.1.23 | A525, G527, G529 | g-j-d |
| 1YVH | cbl e3 ubiquitin protein ligase | ? |  | D229, T231, Y235, E240 | m-o-a-m |
| 1YYQ | trichodiene synthase | ? | 4.2.3.6 | N225, S229, E233 | m-m-o |
| 1ZES | phosphate regulon transcriptional regulatory protein phob | ? |  | D10, D53, M55 | b-f-d |
| **1ZPD** | **pyruvate decarboxylase** | **Mg^2+^** | **4.1.1.1** | **D440, N467, G469** | **k-h-a** |
| **2AKZ** | **gamma enolase** | **Mg^2+^** | **4.2.1.11** | **D244, E292, D317** | **e-h-k** |
| 2AS8 | major mite fecal allergen der p 1 | ? | 3.4.22.- | D56, L57, E59, E91 | d-d-k-k |
| 2B0T | nadp isocitrate dehydrogenase | ? | 1.1.1.42 | D346, D544, D548 | l-m-m |
| **2B82** | **class b acid phosphatase** | **Mg^2+^** | **3.1.3.2** | **D44, D46, D167** | **f-h-b** |
| **2BJI** | **inositol-1(or 4)-monophosphatase** | **Mg^2+^** | **3.1.3.25** | E1070, D1090, I1092 | k-f-g |
|  |  |  |  | **D1090, D1093, D1220** | **f-h-m** |
| 2BVC | glutamine synthetase 1 | ? | 6.3.1.2 | E135, E219, E227 | d-f-d |
|  |  |  |  | E133, H276, E366 | d-d-d |
| 2C31 | oxalyl-coa decarboxylase | ? | 4.1.1.8 | D452, N479, G481 | d-d-d |
| **2C3M** | **pyruvate-ferredoxin oxidoreductase** | **Mg^2+^** | **1.2.7.1** | **D963, T991, V993** | **k-h-a** |
| **2C4N** | **nagd** | **Mg^2+^** |  | **D9, D11, D201** | **f-h-b** |
| 2CW6 | hydroxymethylglutaryl-coa lyase, mitochondrial | ? | 4.1.3.4 | D42, H233, H235, N275 | m-d-f-d |
| 2D0O | diol dehydratase-reactivating factor | ? |  | T105, D166, D183 | d-c-h |
| 2F17 | thiamin pyrophosphokinase 1 | ? | 2.7.6.2 | D46, D71, D73, D100 | j-h-a-k |
| 2HGS | glutathione synthetase | ? | 6.3.2.3 | E144, N146, E368 | d-f-h |
| 3PMG | alpha-d-glucose-1,6-bisphosphate | Mg^2+^ | 5.4.2.2 | S116, D287, D289, D291 | j-f-o-a |

^a^Proteins containing structural motifs are highlighted in bold.
