# Supplementary material for: Discovering structural motifs using a structural alphabet: Application to magnesium-binding sites
Source: BMC Bioinformatics. 2007 Mar 28;8:106. doi: 10.1186/1471-2105-8-106 (PMC1851716; doi:10.1186/1471-2105-8-106)
Supplement: Additional file 2 — 1st-shell patterns common to two Mg2+-proteins. A table listing 1st-shell structural patterns that is common to only 2 Mg2+-binding sites. [file 1471-2105-8-106-S2.doc]

**Additional file 2**. 1^st^−shell patterns occurring in two Mg^2+^−proteins

| Motif ^a^ | PDB code | CATH code^b^ | Functional Group^c^ | EC code^d^ |
| --- | --- | --- | --- | --- |
| h^103−113^l^159-167^d | 1ED9 | 3.40.720.10 | Hydrolase^g^ | 3.1.3.1 |
|  | 1SHQ | 3.40.720.10 | Hydrolase^g^ | 3.1.3.1 |
|  |  |  |  |  |
| j^141−170^f^1^o^1^a | 1WQA | NYC | Isomerase^f^ | 5.4.2.8 |
|  | 3PMG | 3.40.120.10 | Phosphotransferase | 5.4.2.2 |
|  |  |  |  |  |
| k^19−20^f^1^g | 1NUY | 3.30.540.10 | Hydrolase^g^ | 3.1.3.11 |
|  | 12BJI | 3.30.540.10 | Hydrolase^g^ | 3.1.3.25 |
|  |  |  |  |  |
| m^2^c^2^c | 1OFH | − | Hydrolase^g^ | 3.4.25.- |
|  | 1YL7 | 3.40.50.720 | Oxidoreductase^i^ | 1.3.1.26 |
|  |  |  |  |  |
| b^42−43^f^1^d | 1CHN | 3.40.50.2300 | Signal transduction^j^ | − |
|  | 1ZES | 3.40.50.2300 | Transcription activator^j^ | − |
| b^42^d^1^f | 1YIO | NA | DNA-binding^j^ | − |
