# Supplementary material for: Discovering structural motifs using a structural alphabet: Application to magnesium-binding sites
Source: BMC Bioinformatics. 2007 Mar 28;8:106. doi: 10.1186/1471-2105-8-106 (PMC1851716; doi:10.1186/1471-2105-8-106)
Supplement: Additional file 3 — The Ca2+-dataset containing 230 metal-binding sites in 177 nonredundant Ca2+-proteins. A table listing the PDB entries, protein description, native metal-cofactors (if known), EC code, metal-bound amino acid residues, and first-shell structural representation of the 177 nonredundant Ca2+-proteins. [file 1471-2105-8-106-S3.doc]

**Additional file 3**. The Ca^2+^−dataset containing 230 metal−binding sites in 177 nonredundant Ca^2+^−proteins

| **PDB ID** | **Name** | **EC Code** | **Bound Residues** | **1^st^ Shell Struct. Rep.** |
| --- | --- | --- | --- | --- |
| 1AJJ | low-density lipoprotein receptor |  | W22, D25, G27, D29, D35, E36 | g-h-a-b-m-g |
| 1AUI | alpha-lactalbumin | 3.1.3.16 | D30, D32, S34, S36, E41 | m-o-o-a-m |
| 1B9O | alpha-lactalbumin |  | K79, D82, D84, D87, D88 | m-c-f-m-m |
| 1BF2 | isoamylase | 3.2.1.68 | D128, E229, T230, N232, D259 | f-c-d-k-f |
| 1BGP | barley grain peroxidase | 1.11.1.7 | T180, D225, T228, V231, D233 | g-l-m-c-f |
| 1BQB | aureolysin | 3.4.24.27 | D140, D179, D182, L184, E187 | l-m-c-e-l |
|  |  |  | D179, D179, E181, D182, E187 | m-b-c-l |
|  |  |  | Y190, T191, T191, K194, D197 | o-g-g-a |
| 1BRW | pyrimidine nucleoside phosphorylase | 2.4.2.2 | G88, L243, A246, E255 | i-h-d-m |
| 1BYF | polyandrocarpa lectin |  | E86, N89, D107, D108 | a-d-d-d |
| 1C3H | 30 kd adipocyte complement-related  protein precursor |  | D190, Q191, D198 | d-d-d |
|  |  |  | N196, V197, D198 | a-c-d |
| 1CB8 | chondroitinase ac | 4.2.2.5 | E405, D407, D416, Y417 | f-l-d-f |
| 1CLC | endoglucanase celd | 3.2.1.4 | E236, N239, I241, D243, D246 | l-c-d-k-m |
|  |  |  | T356, S358, D361, D362, D401 | f-l-m-m-l |
|  |  |  | S520, D523, I525 | m-c-f |
| 1CRU | quinoprotein glucose dehydrogenase | 1.1.99.17 | A269, Y271, D273, E309 | h-a-h-k |
| 1CVR | gingipain r | 3.4.22.37 | E161, H166, D292 | a-m-m |
|  |  |  | D78, F249, E258 | f-k-m |
|  |  |  | V100, D103, Y105, E107 | h-a-d-b |
|  |  |  | E293, E296, H302- | m-m-f |
| 1CXL | cyclodextrin-glycosyltransferase | 2.4.1.19 | D27, N29, N32, N33, G51, D53 | f-l-p-c-i-f |
|  |  |  | N139, I190, D199, D199, H233- | b-m-d-p |
| 1DV8 | asialoglycoprotein receptor 1 |  | D215, D242, E252, D253 | f-p-f-b |
|  |  |  | Q239, D241, E252, N264, D265 | a-k-f-d-d |
|  |  |  | V190, E196, E277 | d-m-d |
| 1DYK | laminin alpha |  | D2808, L2825, I2874, D2876 | c-h-d-d |
| 1E43 | alpha-amylase | 3.2.1.1 | N102, D194, D194, D200, H235 | b-b-d-p |
|  |  |  | D159, A181, D183, D202, D204 | b-c-d-f-b |
|  |  |  | G300, Y302, H406, D407, D430 | m-c-k-l-b |
| 1EA7 | erine protease |  | D287, I288, A295, G297, D299 | m-m-d-h-a |
|  |  |  | T214, D217, V219, Q221, D224 | f-g-n-p-e |
|  |  |  | N29, E49, D98 | c-k-m |
| 1EE6 | pectate lyase | 4.2.2.2 | D80, V81, K103 | d-c-i |
| 1EGZ | endoglucanase z | 3.2.1.4 | G121, D158, N161 | m-m-c |
| 1G0H | inositol monophosphatase | 3.1.3.25 | E65, D81, D84, D201 | k-f-h-m |
|  |  |  | E65, D81, I83- | k-f-g |
| 1G87 | endocellulase 9g | 3.2.1.4 | D500, E503, N578, N581, D582 | d-k-b-k-l |
|  |  |  | S209, D212, D213, D259 | f-m-m-l |
| 1G8I | neuronal calcium sensor 1 |  | D73, N75, D77, R79, E84 | m-o-o-a-m |
| 1G9G | cellulase cel48f | 3.2.1.4 | Q185, Q185, E190, D405 | f-l-l |
| 1GA6 | serine-carboxyl proteinase | 3.4.23.37 | D328, V329, G344, G346, D348 | d-f-e-i-f |
| 1GCA | glucose/galactose-binding protein |  | D134, N136, D138, K140, Q142, E205 | m-o-o-a-d-b |
| 1GEN | gelatinase a |  | D476, D521, D569, D618 | b-b-b-b |
| 1GGZ | calmodulin-related protein nb-1 |  | D20, D22, D24, C26, E31 | m-o-o-a-m |
| 1GK9 | penicillin g acylase alpha subunit | 3.5.1.11 | D73, V75, D76, P205, D252 | a-b-d-e-a |
| 1GU6 | cytochrome c552 |  | E215, Y216, K261, Q263 | d-d-d-h |
| 1GUI | laminarinase 16a |  | T8, G47, D150 | o-i-h |
| 1H5V | endoglucanase 5a | 3.2.1.4 | G127, D166, N168, N169- | m-m-m-b |
| 1H6L | phytase |  | E43, D308, N339, I340, D341- | c-c-c-e-e |
|  |  |  | D308, G309, N336, E338 | c-d-f-d |
|  |  |  | D56, P57, V101- | d-c-c |
|  |  |  | D258, E260, Q279- | d-d-k |
| 1HT6 | alpha-amylase isozyme 1 | 3.2.1.1 | N92, D139, D139, A142, D149 | c-n-a-c-b |
|  |  |  | D128, D143, F144, A147, D149 | k-c-c-b-c |
| 1HX0 | alpha amylase (ppa) | 3.2.1.1 | N100, R158, D167, D167, H201 | d-f-p-b |
| 1HYO | fumarylacetoacetate hydrolase | 3.7.1.2 | D126, E199, E201, D233 | d-d-d-d |
| 1I40 | inorganic pyrophosphatase | 3.6.1.1 | D65, D70, D102- | i-o-f |
| 1I8A | endo-1,4-beta-xylanase a | 3.2.1.8 | D81, N83, E91, D93, D94 | f-o-f-l-c |
|  |  |  | V10, D12, E14, D16, E130 | d-h-a-f-d |
|  |  |  | D60, V62, D74, D154, A155 | f-g-c-d-d |
| 1IAG | adamalysin ii | 3.4.24.46 | E9, D93, C197, N200 | d-b-l-g |
| 1IC6 | proteinase k | 3.4.21.64 | P175, V177, D200 | k-c-b |
| 1IME | inositol monophosphatase | 3.1.3.25 | E70, D90, I92- | k-f-g |
| 1IVG | influenza a subtype n2 neuraminidase | 3.2.1.18 | D293, G297, G345, Q347 | f-f-g-c |
| 1J1N | algq2 |  | D171, N173, N175, K177, D179, E180 | p-o-o-a-b-c |
| 1J1T | lysozyme | 3.2.1.17 | D106, D114, V116, N119, I121 | d-o-g-o-a |
| 1J9L | stationary phase survival protein |  | D8, D9, S39, N95 | k-b-h-e |
| 1JTG | beta-lactamase inhibitory protein |  | D133, D135, R144 | f-l-d |
| 1JZN | galactose-specific lectin |  | Q96, D98, E104, N119, D120 | a-k-a-d-d |
| 1K12 | lectin |  | N35, D38, N40, S49, C146, E147 | m-o-a-a-b-d |
| 1K94 | grancalcin |  | D132, D134, S136, T138, E140, E143 | mo-o-a-f-m |
| 1K96 | s100a6 |  | S20, E23, D25, T28, E33 | m-o-f-c-m |
| 1K9U | polcalcin phl p 7 |  | D13, N15, D17, K19, E24 | m-o-o-a-m |
| 1KA1 | halotolerance protein hal2 | 3.1.3.7 | E72, D142, I144 | b-f-g |
| 1KFQ | phosphoglucomutase 1 | 5.4.2.2 | S126, D308, D310, D312 | j-f-o-a |
| 1KP4 | phospholipase a2 | 3.1.1.4 | D43, L44, D65 | h-j-m |
| 1KU0 | l1 lipase | 3.1.1.3 | G286, E360, D365, P366 | i-m-g-c |
| 1KV9 | type ii quinohemoprotein  alcohol dehydrogenase | 1.1.99.- | E173, N250, D295- | n-d-p |
| 1L6R | hypothetical protein ta0175 |  | D8, D174, N177, D178 | f-b-l-m |
| 1L7L | pa-i galactophilic lectin |  | Y36, D100, T104, N107, N108 | k-d-l-g-g |
| 1LE6 | group x secretory phospholipase a2 | 3.1.1.4 | F26, G28, G30, D47, D47 | g-g-i-m |
| 1LED | west-central african legume lectin iv |  | D131, W133, N135, D140 | b-c-k-f |
| 1LWJ | 4-alpha-glucanotransferase | 2.4.1.25 | D13, N15, D17, V19, D21 | f-o-o-a-f |
| 1M1U | integrin alpha-m |  | S142, S144, T209- | f-l-d |
| 1M56 | cytochrome c oxidase | 1.9.3.1 | E54, E54, A57, G59- | m-g-i |
| 1MHO | s-100 protein |  | S18, E21, D23, K26, E31 | m-o-f-cm |
| 1MNZ | xylose isomerase | 5.3.1.5 | E217, H220, D255, D257 | d-l-d-k |
| 1MXG | alpha amylase | 3.2.1.1 | N110, D155, G157, D164, G202 | b-I-I-c-l |
| 1N28 | phospholipase a2,  membrane associated | 3.1.1.4 | H27, G29, G31, D48 | g-e-i-m |
|  |  |  | F23, G25, Y112 | g-j-c |
| 1NBC | cellulosomal scaffolding protein a |  | T44, T44, D46, T122, N125, D126 | e-i-f-p-f |
| 1NBW | glycerol dehydratase reactivase |  | T104, T104, D168, D185, E31- | d-c-h |
| 1NKG | rhamnogalacturonase b | 4.2.2.- | E347, D349, Q351, D502 | d-h-a-b |
| 1NLS | concanavalin a |  | D10, Y12, N14, D19 | f-d-f-a |
| 1NPS | development-specific protein s |  | Y7, T37, S39, N76 | b-b-b-i |
|  |  |  | N36, Q53, S79 | k-b-b |
| 1NSC | neuraminidase | 3.2.1.18 | D292, T296, D323, G343, G345 | f-m-d-g-c |
| 1O4Y | beta-agarase a | 3.2.1.81 | D22, S47, N49, S91, D279 | k-h-a-a-b |
| 1O88 | pectate lyase | 4.2.2.2 | D129, D131, E166, D170 | p-f-c-c |
| 1O9I | pseudocatalase | 1.11.1.6 | D57, D61, N218, S220, G222- | f-f-l |
| 1OAH | cytochrome c nitrite reductase | 1.7.2.2 | E236, Y237, K296, Q298- | d-d-d-h |
|  |  |  | G75, E114, T115 | j-d-d |
| 1OBR | carboxypeptidase t | 3.4.17.18 | D56, E57, E61, E104 | c-d-d-c |
|  |  |  | S50, D51, D51, E57, E59 | e-h-d-d |
|  |  |  | D51, E59, N101- | h-d-n |
|  |  |  | S7, Y9, E14- | m-c-m |
| 1OD3 | putative xylanase |  | Q30, E32, S52, D142 | f-l-d-b |
| 1OFL | chondroitinase b | 4.2.2.4 | N213, E243, E245- | b-b-b |
| 1OH4 | beta-mannosidase |  | D12, G44, G46, D165 | h-o-a-h |
| 1OMR | recoverin |  | D110, D112, N114, T116, E121 | m-o-o-a-m |
| 1OS1 | phosphoenolpyruvate carboxykinase | 4.1.1.49 | K213, H232, D269 | m-d-b |
| 1OYG | levansucrase | 2.4.1.10 | D241, Q272, L308, N310, D339 | a-d-m-c-b |
| 1PJX | diisopropylfluorophosphatase | 3.1.8.2 | E21, N120, N175, D229 | h-d-h-e |
|  |  |  | D232, L273, H274- | d-b-d |
| 1PK6 | complement c1q subcomponent |  | Q177, D172, Y173, Q179- | d-d-d |
| 1POA | phospholipase | 3.1.1.4 | Y27, G29, G31, D48 | g-g-i-m |
| 1PWB | pulmonary surfactant-associated  protein d |  | E321, N323, E329, N341, D342 | a-h-c-d-d |
|  |  |  | D297, E301, D324, E329, D330 | k-h-g-c-b |
| 1Q6Z | benzoylformate decarboxylase | 4.1.1.7 | D428, N455, T457- | k-h-a |
| 1QLA | fumarate reductase flavoprotein subunit | 1.3.99.1 | S371, M372, G373, E393, A395 | f-b-l-k-c |
| 1QMD | phospholipase c | 3.1.4.3 | D269, G271, D336, A337 | d-f-l-h |
|  |  |  | D293, N294, G296, D298 | k-c-d-d |
|  |  |  | T272, D273, N297, D298 | c-b-g-h |
| 1R13 | pulmonary surfactant-associated  protein a |  | E195, N214, D215 | a-d-d |
| 1R17 | fibrinogen-binding protein sdrg |  | E292, S297, I300, E307 | f-g-a-a |
| 1R55 | adam 33 | 3.4.24.- | E213, D296, C404, N407 | d-b-l-g |
| 1R64 | kexin | 3.4.21.61 | D277, D320, E350- | e-a-c |
|  |  |  | D135, D184, K224, N227, F229, G231 | f-l-e-o-ad |
| 1R6V | subtilisin-like serine protease |  | E137, D179, K219, D221, K223, I225 | a-l-c-h-a-d |
| 1RLW | phospholipase a2 | 3.1.1.4 | D40, T41, D43, N65 | g-c-d-i |
| 1RRO | rat oncomodulin |  | D90, D92, D94, K96, E101 | m-o-o-a-m |
| 1RU4 | pectate lyase | 4.2.2.2 | D51, D53, S55, Y57, E62 | f-l-m-c |
|  |  |  | D209, D233, D234, D237 | p-h-d-e-c |
| 1S1D | apyrase | 3.6.1.5 | S98, E145, E214, S275 | b-b-b-b |
| 1SAC | serum amyloid p component |  | D58, N59, E136, Q137, D138 | h-k-c-f-b |
|  |  |  | E136, D138, Q148- | c-b-p |
| 1SNC | thermonuclease precursor | 3.1.31.1 | D21, D40, T41 | b-c-d |
| 1SRA | sparc |  | D222, P225, D227, Y229, E234 | m-c-h-a-m |
|  |  |  | D257, D259, D261, Y263, E268 | k-g-l |
| 1SRR | sporulation response regulatory protein |  | D11, D54, K56- | b-f-d |
| 1SU4 | sarcoplasmic/endoplasmic reticulum  calcium ATPase 1 | 3.6.3.8 | V304, A305, I307, E309, N796, D800 | m-m-p-h-m-m |
|  |  |  | N768, E771, T799, D800, E908 | m-m-m-m-m |
| 1SZB | mannose binding lectin-associated  serine protease-2 related protein, map19 |  | D123, I124, E126, N143, H144, G147 | d-d-k-d-e-a |
|  |  |  | E52, D60, D105, S107, N108 | d-c-f-d-f |
| 1T1G | kumamolisin |  | D316, I317, G334, G336, D338 | m-m-d-km |
| 1THM | thermitase | 3.4.21.66 | D5, D47, V82, N85, T87, I89 | f-l-e-o-a-d |
| 1TKJ | aminopeptidase | 3.4.11.- | D3, I4, D262, D266 | I-I-m-m |
| 1TL9 | calpain 1, large [catalytic] subunit | 3.4.22.52 | V99, G101, D106, E185 | e-a-p-c |
|  |  |  | E302, D309, M329, D331, E333 | d-k-d-h-a |
| 1TN3 | tetranectin |  | D116, E120, G147, E150, N151 | k-h-j-c-c |
|  |  |  | Q143, E150, D165 | c-c-d |
| 1TRK | transketolase | 2.2.1.1 | D157, N187, I189 | k-h-a |
| 1UA7 | alpha-amylase | 3.2.1.1 | N101, T137, D146, H180 | b-m-d-p |
| 1UHN | calcineurin b-like protein 2 |  | S58, I62, D64, L66, E71 | m-c-h-am |
| 1ULV | glucodextranase | 3.2.1.70 | L139, T142, G144, D146, D159 | h-a-h-a-d |
|  |  |  | F358, Q362, E409- | m-c-m |
|  |  |  | D136, P137, N211, I212 | d-f-d-d |
|  |  |  | V946, A969, D980, D982, D985 | d-c-a-l-c |
|  |  |  | D782, P783, D807, L808 | d-e-c-f |
|  |  |  | D782, D786, V991, Q999 | d-l-c-k |
| 1UOW | synaptotagmin i |  | M302, D303, D363, D365 | b-e-d-f |
|  |  |  | D303, D309, D363, D363, Y364, D365 | e-d-d-d-f |
| 1UP8 | vanadium-dependent bromoperoxidase 1 |  | F360, Q362, D364, D367, D367, Q369 | i-c-k-o-a |
| 1UR4 | galactanase | 3.2.1.89 | D272, D274, H276, N278, S367, D370 | f-o-a-f-f-m |
| 1USR | hemagglutinin-neuraminidase  glycoprotein | 3.2.1.18 | D261, S264, V266, V296 | m-m-l-b |
| 1UV4 | arabinan-endo 1,5-alpha-l-arabinase | 3.2.1.99 | D77, E135, D257 | d-d-c |
| 1UYY | cellulase b |  | Q7, E9, K29, N123 | f-l-d-b |
|  |  |  | G16, Y33, D35, D38 | i-i-e-a |
| 1UZJ | fibrillin-1 |  | D1487, V1488, E1490, N1504, T1505, S1508 | d-d-k-d-e-a |
| 1V3E | hemagglutinin-neuraminidase  glycoprotein | 3.2.1.18 | D279, S282, G284, A316 | m-g-i-b |
| 1V73 | psychrophilic phosphatase i | 3.1.3.48 | D78, H80, D114- | k-o-k |
| 1VBL | pectate lyase 47 | 4.2.2.2 | D190, D229, D233 | f-c-c |
| 1VCL | hemolytic lectin cel-iii |  | D23, I24, G26, D43 | e-h-l-m |
|  |  |  | D121, I122, G124, D141 | e-h-l-p |
| 1VEM | beta-amylase | 3.2.1.2 | E56, D60, E141, E144- | m-h-m-m |
| 1W0N | endo-1,4-beta-xylanase | 3.2.1.8 | E16, E18, D35, D125, D125 | c-f-b-d |
| 1W0P | aminomethyltransferase | 2.1.2.10 | A253, N256, D289, T313 | f-c-c-k |
|  |  |  | P548, D621, D682, A683 | a-d-d-d |
| 1WAD | cytochrome |  | D11, I13, N19, L20 | f-a-c-d |
| 1WC5 | adenylate cyclase | 4.6.1.1 | D1017, I1018, D1061 | d-e-a |
| 1WMD | protease | 3.4.21.- | E186, S194, D197, H201 | d-o-l-k |
|  |  |  | D384, T386, P388, D391, N392 | h-a-d-b-l |
|  |  |  | D367, L368, D369, D394, E400 | b-d-c-c-c |
| 1WY9 | allograft inflammatory factor 1 |  | S94, S97, T100 | c-f-c |
| 1WZL | alpha-amylase ii |  | N143, D145, N148, D149, G169, D171 | f-l-p-c-i-f |
| 1X1J | xanthan lyase | 4.2.2.12 | D515, D516, E517, E676 | k-b-c-f |
| 1X8L | retinol dehydratase |  | E118, N121, D122 | m-m-m |
| 1Y1X | leishmania major homolog of  programmed cell death 6 protein |  | D37, D39, S41, A43, E48 | m-o-o-a-m |
| 1Y4J | sulfatase modifying factor 2 |  | N194, L195, D208, F210 | c-d-o-a |
|  |  |  | D229, L230, G232, V234 | k-g-h-a |
| 1Y7B | beta-xylosidase, family 43 glycosyl hydrolase | 3.2.1.37 | N333, G362, D528- | h-i-b |
| 1Y93 | macrophage metalloelastase | 3.4.24.65 | D158, G190, G192, D194- | b-k-c |
| 1Y9Z | alkaline serine protease | 3.4.21.- | E2, D39, I76, N78, E80, V82 | e-h-e-i-f |
| 1YDY | glycerophosphoryl diester  phosphodiesterase | 3.1.4.46 | E63, D65, E171 | d-d-d |
| 1YII | annexin a5 |  | M28, G30, G32, E72 | g-g-i-m |
| 1YS6 | transcriptional regulatory protein prra |  | D15, D58, N60- | b-f-d |
| 1YYD | carbonic anhydrase ii | 4.2.1.1 | S174, D191, T193, T196, D198 | n-f-l-c-f |
|  |  |  | D47, G62, D64, S66 | m-j-o-m |
| 1Z3U | angiopoietin-2 |  | D429, D431, C433, C435 | c-f-f-l |
| 1ZED | alkaline phosphatase | 3.1.3.1 | E216, F269, E270, D285 | l-b-f-c |
| 1ZH2 | kdp operon transcriptional  regulatory protein kdpe |  | E8, D9, D52, G54 | f-b-f-d |
| 2AEF | calcium-gated potassium channel mthk |  | D184, D184, E210, E212 | d-d-b |
| 2AYH | 1,3-1,4-beta-d-glucan | 3.2.1.73 | P9, G45, D207- | h-p-b |
| 2AZ1 | nucleoside diphosphate kinase | 2.7.4.6 | A134, F137, D138- | m-c-f |
| 2B59 | cog1196: chromosome segregation  ATPases |  | D135, N137, N139, A141, D146 | m-o-o-a-m |
|  |  |  | D102, D106, S108, D113 | l-h-a-m |
| 2BF6 | exo-alpha-sialidase | 3.2.1.18 | D296, D298, D319, Y320 | d-d-c-e |
| 2BIB | eichoic acid phosphorylcholine esterase/  choline binding protein |  | E36, D37, D39 | a-f-d |
|  |  |  | D37, G92, D95, E96 | f-m-m-m |
| 2BL0 | major plasmodial myosin heavy chain | 3.6.1.32 | D15, D17, D19, K21 | m-o-o-a-m |
| 2BOQ | versatile peroxidase vpl2 |  | D48, G60, D62, S64 | m-j-j-o |
|  |  |  | S170, D187, T189, V192, D194 | m-i-f-b-c |
| 2BQ4 | basic cytochrome c3 |  | D10, L12, E20, Y21 | f-c-c-c |
| 2BV2 | ciona betagamma-crystallin |  | E7, I33, S35, D75 | b-a-b-k |
|  |  |  | D32, D48, E76, E76, S78 | k-b-b-b |
| 2BWR | sathyrella velutina lectin |  | D343, T345, D347, C349, D351 | f-o-o-a-d |
| 2C1V | di-haem cytochrome c peroxidase | 1.11.1.5 | N93, T270, P272- | c-p-e |
| 2CBL | proto-oncogene cbl |  | D229, T231, N233, Y235, E240 | m-o-o-a-m |
| 2CCM | calexcitin |  | D23, N25, D27, V29, D34 | m-o-o-a-m |
|  |  |  | D74, N76, D78, Q80, E85 | m-o-o-a-m |
| 2FI1 | hydrolase,  haloacid dehalogenase-like family |  | D12, G14, D160 | f-h-b |
| 2MAS | inosine-uridine nucleoside  n-ribohydrolase | 3.2.2.1 | D10, D15, D15, T126, D242- | d-m-b-l |
| 2POR | porin |  | D93, D95, N100, D101- | p-k-a-d |
| 2PSR | psoriasin |  | D62, N64, D66, K68, E7-3- | m-o-o-a-m |
| 2SAS | sarcoplasmic Ca-binding protein |  | D19, N21, D23, S25, D30- | m-o-o-a-m |
| 2SCP | sarcoplasmic Ca-binding protein |  | D16, D18, D20, A22, D27 | m-o-o-a-m |
| 5CHY | chey |  | D13, D57, N59- | b-f-d |
| 5ENL | enolase | 4.2.1.11 | D246, E295, D320 | f-h-k |
| 7TAA | taka amylase | 3.2.1.1 | N121, E162, D175, H210 | b-m-d-p |
